# 512-Channel Geometric Droplet-Splitting Microfluidic Device by Injection of Premixed Emulsion for Microsphere Production

**DOI:** 10.3390/polym12040776

**Published:** 2020-04-01

**Authors:** Chul Min Kim, Hye Jin Choi, Gyu Man Kim

**Affiliations:** 1 Department of Mechanical Engineering, Korea Polytechnic University, Siheung-Si 15073, Korea; faithfulsaint@daum.net; 2 School of Mechanical Engineering, Kyungpook National University, Daegu 41566, Korea; hyejin0058@gmail.com

**Keywords:** premixed emulsion, droplet-splitting, microfluidics, microsphere, high throughput

## Abstract

We present a 512-channel geometric droplet-splitting microfluidic device that involves the injection of a premixed emulsion for microsphere production. The presented microfluidic device was fabricated using conventional photolithography and polydimethylsiloxane casting. The fabricated microfluidic device consisted of 512 channels with 256 T-junctions in the last branch. Five hundred and twelve microdroplets with a narrow size distribution were produced from a single liquid droplet. The diameter and size distribution of prepared micro water droplets were 35.29 µm and 8.8% at 10 mL/h, respectively. Moreover, we attempted to prepare biocompatible microspheres for demonstrating the presented approach. The diameter and size distribution of the prepared poly (lactic-co-glycolic acid) microspheres were 6.56 µm and 8.66% at 10 mL/h, respectively. To improve the monodispersity of the microspheres, we designed an additional post array part in the 512-channel geometric droplet-splitting microfluidic device. The monodispersity of the microdroplets prepared with the microfluidic device combined with the post array part exhibited a significant improvement.

## 1. Introduction

Microspheres have been used as carriers to deliver functional materials such as drugs, proteins, and chemicals [1,2]. In the delivery of functional materials, a controlled release profile is necessary to control the functional materials quantitatively [3]. Size distribution of the micro carrier is an important factor governing the controlled release of functional materials [4]. Additionally, monodispersed microspheres have potential value for use as particulate blood analogues [5,6,7]. They have been mainly used as microfluidic systems to prepare monodispersed microspheres. Droplet-based microfluidic systems enable us to prepare monodisperse microspheres with precisely controlling the loading of functional materials [8,9]. Despite the promise of microfluidic system, conventional microfluidic systems have the limitations of low production rates and complexity for microsphere preparation [10,11,12].

Many groups have employed various approaches to improve the microsphere productivity of microfluidic systems, such as the use of parallel systems, multi-nozzle systems, and robust systems [12,13,14,15]. However, these approaches require complicated fabrication processes and precise handling of multilayer systems, as well as additional devices for generating high pressure [14,15]. Thus, it is not easy to implement them for scale up for industry application because of low reproducibility owing to a complicated fabrication process and complex systems.

Therefore, symmetrical droplet splitting using a microchannel geometry can be considered an alternative solution to the problem [16]. Multiple droplets can be generated from a single liquid plug through symmetric droplet splitting in a geometric T-junction microchannel with narrow size distribution [16]. Weitz et al. first described geometric droplet splitting by using a T-junction composed of 8 channels [17]. Our group proposed 512 channel T-junction passive breakup devices for preparing microspheres, which generate monodispersed chitosan microspheres at 42.7 kHz, but its microsphere production rate required improvement for mass production [18].

To overcome this limitation, we can consider the injection of premixed emulsion into a microfluidic device as an alternative approach [19,20]. With this approach, the size distribution of the microspheres was not more monodispersed than the size distribution of the microspheres prepared by injecting the dispersed and continuous phases independently. However, this approach of injecting premixed emulsion proved to be beneficial in terms of mass production and the one-step preparation process [19,20]. Especially, a design based on a single micro channel can drastically decrease flow resistance, which is the most important factor governing the production of microspheres at high flow rates. Additionally, this microfluidic device only required conventional photolithography and PDMS casting for fabrication, without any additional fabrication processes or additional devices. Moreover, this microsphere production process is a single-step process, which makes it suitable for mass producing microspheres.

In this study, we present a mass production method of a microsphere with a 512-channel geometric droplet-splitting microfluidic device by injecting a premixed solution. The performance of the fabricated is measured in W(Water)/O(Oil) system with an inverted microscope and the Image J software program. To demonstrate the proposed approach, the fabricated microfluidic device is used to prepare PLGA (poly (lactic-co-glycolic acid)) microspheres, which is a biocompatible and biodegradable co polymer approved by FDA (the Food and Drug Administration) [17]. The morphology of the prepared PLGA microspheres is inspected with scanning electron microscopy (SEM). Furthermore, the additional post array part in the microfluidic device is designed to improve size distribution of microspheres. Three types of post array parts are evaluated in terms of droplet splitting and size distribution.

## 2. Materials and Methods

### 2.1. Materials

Polydimethylsiloxane (PDMS, Sylgard 184, Midland, MI, USA) solution was purchased from Dow corning. PLGA (MW: 40,000~75,000, 65:35), dimethyl carbonate (DMC, anhydrous, 99%), polyvinyl alcohol (PVA, MW: 31,000~50,000, hydrolyzed), Mineral oil and SPAN^®^80, Hexadecane were obtained from Sigma-Aldrich (St. Louis, MO, USA). SU-8 2050 and SU-8 developer were purchased from MicroChem (Round Rock, TX, USA).

### 2.2. Design of Microfluidic Device

**Figure 1:**
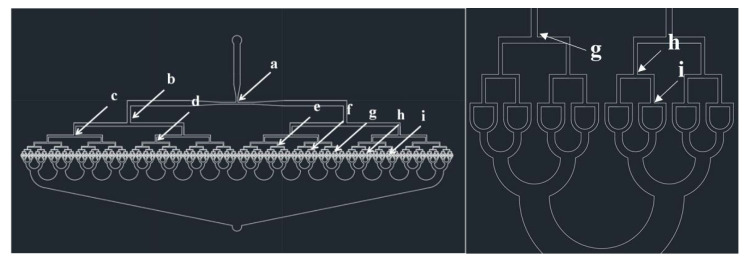
CAD design of 512-channel geometrical droplet-splitting microfluidic device.

**Figure 2:**
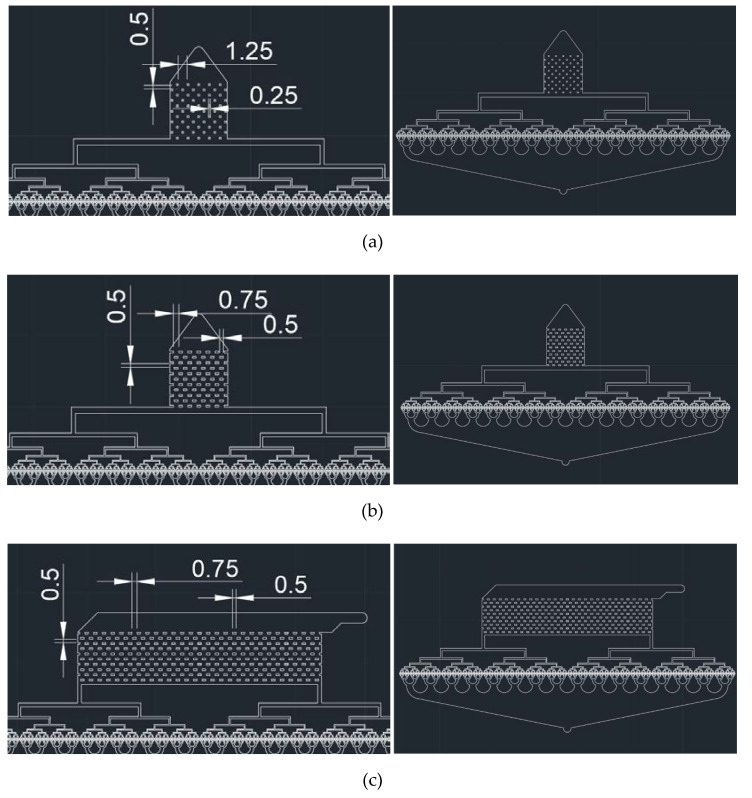
CAD designs of post array part for 512-channel geometric droplet-splitting microfluidic device: (**a**) type a, (**b**) type b, and (**c**) type c (unit: mm).

Figure 1 shows a CAD (Computer-Aided design, AutoCAD, San Rafael, CA, USA) design of the 512-channel geometric droplet-splitting microfluidic device. Five hundred and twelve micro channels were prepared from a single microchannel by ninth division of the microchannel. The widths of the microchannels at T-junctions gradually decreased for minimization of flow resistance and stable microsphere preparation (Table 1). The 512 channels were merged into a single microchannel by sixth combining micro channels to prevent merging of microdroplets in the collection part. The post array part was designed to adequate droplet-splitting time as shown in Figure 2.

Three types of post array parts were designed to achieve stable microdroplet preparation. Type 1 post array had dimensions of 250 μm × 250 μm with a horizontal interval of 1250 µm. Type 2 post array had dimensions of 500 μm × 250μm with a horizontal interval of 750 µm. Both designs had the same dimensions, except for the width and horizontal interval of the post arrays.

In case of the Type 1 and Type 2 post array parts, the concentrations of droplets along the streamline were found as the flow rate increased. To prevent the phenomena in the Type 3 post array, the inlet direction was designed to be perpendicular to the outlet direction. The width and horizontal interval of the Type 3 post array were equal to those of the Type 2 post array.

### 2.3. Fabrication of 512-Channel Geometric Droplet-Splitting Microfluidic Device

The master mold of microfluidic device with 40 μm thickness is fabricated by conventional photolithography. A negative photoresist (SU-8 2050, Kayaku Advanced Materials, Inc., Westborough, MA, USA) is dispensed and spread on the silicon wafer with two different speeds (500 rpm for 5 s and 4000 rpm 30 s). The spin coated master mold is baked at 65 °C for 3 min and 95 °C for 5 min. The photoresist is exposed ultraviolet (UV) radiation through transparency mask at 10 mW/cm^2^ for 25 s. The Post exposure baking process is conducted at 65 °C for 5 min and 95 °C for 12 min. The exposed wafer mold is immersed in SU-8 developer for 15 min to remove uncured photoresist. Finally, the wafer mold was rinsed with Isopropyl alcohol and dried with nitrogen gas.

To prepare PDMS solution, PDMS prepolymer is mixed with curing agent at a ratio of 10:1. The resulting solution is poured onto the fabricated master and degassed in the vacuum chamber to remove bubbles. The PDMS solution poured master mold is heated in an oven at 90 °C for 2 h. The cured PDMS block is detached carefully from the master mold and boned with slide glass by plasma treatment.

### 2.4. Experimental Setup of Microfluidic System

Microdroplets were prepared by injecting a premixed solution into the fabricated microfluidic system with a syringe pump (Harvard Apparatus, Pump 11 Elite & Pico Plus, Holliston, MA, USA). The syringe is connected to the microfluidic device through a tygon tube or Teflon tube according to the properties of the polymer solution. The behavior of microdroplets in the microchannel is observed using an inverted optical microscope (CSB-IH5, Samwon Scientific Ind. Co., Ltd., Seoul, South Korea). The diameter and size distribution of microspheres are measured by the ImageJ software program (National Institutes of Health, Bethesda, MD, USA). The morphology of prepared microspheres is observed with SEM.

### 2.5. Preparation of Microspheres Using 512-Channel Geometric Droplet-Splitting Microfluidic Device

**Figure 3:**
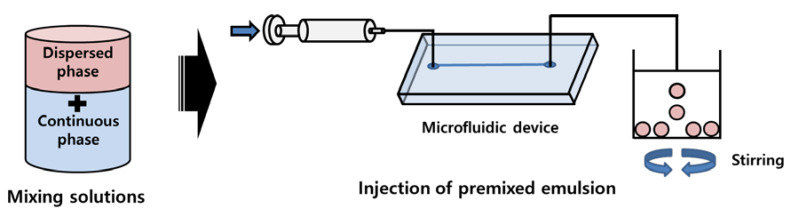
Microsphere preparation process of 512-channel geometric droplet-splitting microfluidic device by injecting premixed emulsion solution.

Figure 3 shows the microdroplet preparation process of 512-channel geometric droplet-splitting microfluidic device by injection of premixed emulsion solution. First, deionized water and mineral oil (with 3 wt.% SPAN 80) are prepared as the dispersed phase and continuous phase, respectively. The two solutions are gently mixed and injected into the device using the syringe pump. The fabricated microfluidic device was bonded with a slide glass by plasma treatment. The assembled microfluidic device was placed in an oven at 90 °C to recover surface hydrophobicity for W/O system. In order to prepare PLGA microspheres, PLGA powders were dissolved in DMC solution to prepare 2 wt.% PLGA solution as the dispersed phase. 2 wt.% PVA solution is prepared as the continuous phase by dissolving PVA in deionized water at 90 °C. The PDMS microfluidic device was bonded with a slide glass by plasma treatment. After plasma treatment, PVA coating was applied in the microchannel to maintain its hydrophilic condition for stable microdroplet preparation. The 2 wt.% PLGA solution and 1 wt.% PVA solution were gently mixed in the ratio of 0.75:1. The premixed emulsion was injected into the fabricated microfluidic device by using a syringe pump. The prepared PLGA microspheres were collected in the 2 wt.% PVA solution under stirring at 300 RPM for 5 h to remove solvent from the microspheres. In order to remove satellites, the sieve was used. The mesh size of the sieve was determined according to the microsphere size. The satellites had fallen through the sieve and the prepared microspheres were collected in the sieve.

## 3. Results and Discussion

For stable preparation of the proposed device, optimization of the mixing ratio for the dispersed phase and the continuous phase is necessary. As the volume of the dispersed phase increased, the size of the prepared microdroplet increased, and the microdroplets aggregated because the distance between the microdroplets decreased. Moreover, when the microdroplets aggregated, the flow rate was changed periodically, resulting in unstable microdroplet preparation. By contrast, as the volume of the continuous phase increased, the microdroplet production rate decreased, resulting in ineffective microdroplet preparation.

**Figure 4:**
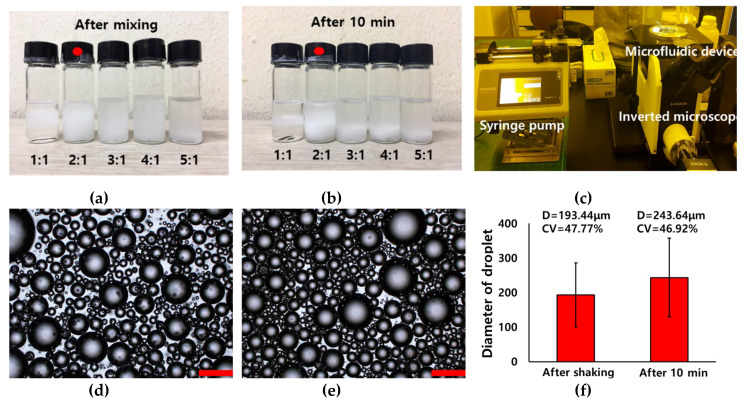
Pictures of dispersion test about ratio of water in mineral oil (3wt.% span 80): (**a**) after shaking and (**b**) 10 min, (**c**) microfluidic system with premixed emulsion. Optical images (**d**,**e**) and graph (**f**) of inherent droplet after shaking and 10 min.

Therefore, a dispersion test of the premixed emulsion solution was conducted to determine the suitable mixing ratio for the premixed emulsion solution. The dispersed phase and continuous phases were gently mixed and placed at room temperature for 10 min. The dispersion condition of the premixed emulsion solution was observed after mixing and 10 min (Figure 4a,b). As a result, the optimized mixing ratio of premixed emulsion solution was 2(mineral oil):1(water). Droplets in premixed emulsion were observed by microscope after mixing and 10 min (Figure 4d,e). The droplet sizes were 193.44 and 243.64 μm with polydisperse size distribution (CV = 47.77, 46.92%) after mixing and 10minutes as shown Figure 4f. The droplet size after 10 min was larger than that after mixing, owing to aggregation with each other. The prepared premixed emulsion was injected into a single inlet of the microfluidic device by using a syringe pump as shown Figure 4c.

**Figure 5:**
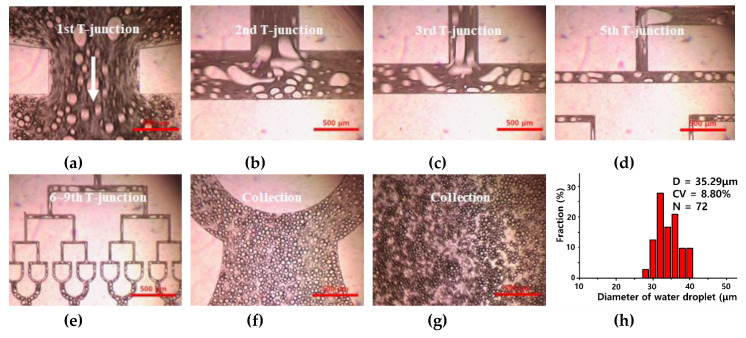
Optical images of water droplets prepared using 512-channel geometric droplet-splitting microfluidic device using premixed emulsion solution at (**a**) 1st, (**b**) 2nd, (**c**) 3rd, (**d**) 5th, and (**e**) 6–9th T-junctions, and (**f**,**g**) collection part. (**h**) The graph of diameter and size distribution for the prepared micro water droplet (Q_mix_ = 10 mL/h).

Figure 5a–e shows optical images of the preparation of water microdroplets with the microfluidic device by injection of premixed emulsion solution. The prepared water droplets of various sizes flowed stably into the first T-junction part of the 512-channel geometric droplet-splitting microfluidic device. The droplets at T-junction were contracted and expanded with fluid flow due to their large volume. When the microdroplet enters at the T-junction, it is contracted and elongated along the streamline.

If the prepared microdroplets were large enough to be split, they were divided into two droplets at a T-junction. By contrast, if the prepared microdroplets were not large enough to be split, they were passed through the T-junction without splitting. As the width of the microchannel of T-junction decreased, smaller water microdroplets could be divided. The prepared microdroplets were gathered at a collection region without merging (Figure 5f,g).

However, many satellites were found during the splitting process. The satellites were separated from the parent droplet by the imbalance of capillary force during droplet formation [21]. Because the microsphere with satellites have a broad size distribution, it is better not to generate satellites. However, formation of satellites was unavoidable, even with the microfluidic system for higher flow rate [22,23,24,25,26]. Furthermore, the presented method is a combination of the conventional emulsification method and microfluidic method. Therefore, removing satellites from microspheres is necessary. The number of satellites significantly decreased or were removed when a viscosity of dispersed phase was higher than viscosity of the continuous phase [27]. In this case, the satellites were filtered during the collection process, therefore they are ignored during the measuring process.

The diameter and size distribution (C.V. (Coefficient Variation)) of the prepared water microdroplets were 35.29 µm and 8.8% when Q_mix_ was 10 mL/h (Figure 5h). Fine water microdroplets could be prepared stably and repeatably. Based on this result, we attempted to prepare a biocompatible PLGA microsphere with the fabricated microfluidic device. Firstly, a dispersion test of the DMC solution with the PVA solution was conducted to find an optimized mixing ratio for a premixed emulsion solution.

**Figure 6:**
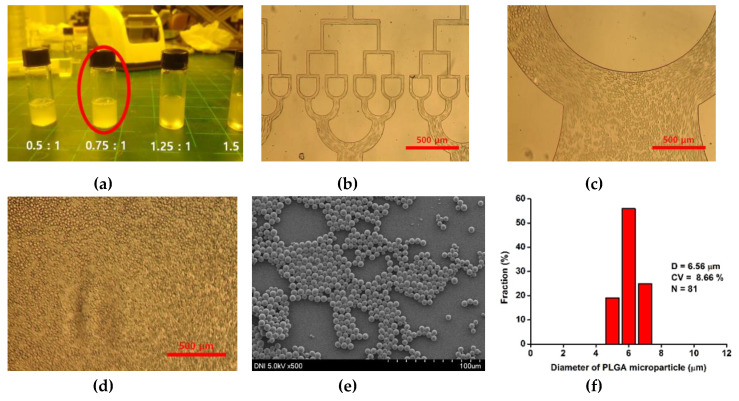
(**a**) Dispersion test about the ratio of DMC solution with PVA solution, (**b**–**d**) optical images of PLGA micro droplet in microchannel, (**e**) SEM image and (**f**) diameter and size distribution of PLGA microsphere prepared by using injecting premixed emulsion at 10 mL/h.

As shown in Figure 6a, when DMC and PVA were mixed in the ratio of 0.75 and 1, the premixed emulsion solution was most balanced and stable for 10 min. PLGA microspheres were prepared at an emulsion flow rate of 10 mL/h. PLGA microspheres with various sizes were formed in the microchannels and split at the T-junctions in the microchannel (Figure 6b–d). The prepared PLGA microspheres were smaller than water microdroplets, owing to the low viscosity of the PVA solution. the PLGA microspheres were collected stably in the final collection part of the 512-channel geometric droplet-splitting microfluidic device. The few satellites were found at collection part because viscosity of the polymer solution is higher than that of the PVA solution, they are filtered during washing process. The prepared PLGA microspheres were overserved with a field-emission scanning electron microscope (Figure 6e). The size distribution and diameter of the prepared PLGA microspheres were 6.56 µm and 8.66% (Figure 6f).

The PLGA microsphere size was similar to that of red blood cells, and they can be considered an RBCs model [28,29]. The size of the PLGA microsphere is controlled by concentration of the PLGA solution and PVA solution [4,30]. The microsphere size is increased with low concentration of PVA solution and high concentration of the PLGA solution.

Weitz’s group presented a microfluidic post array device based on droplet-splitting by using obstacles to prepare microspheres with premixed emulsion solution [20]. They prepared photocurable microspheres with a C.V. of 13% by using the microfluidic post array device. A comparison of the results of this study with those of Weitz’s group revealed that the size distribution of microspheres could be improved to 33% by using the proposed 512-channel geometric droplet-splitting microfluidic device because the droplets are guided for symmetrical splitting by using the geometry of T-junction micro channel, as opposed to the irregular and asymmetrical splitting pattern employed in the previous study. We demonstrated the preparation of PGLA microspheres by using the 512-channel geometric droplet-splitting microfluidic device. However, we observed that large microdroplets were formed intermediately because the number of T-junctions was inadequate for complete droplet splitting in some cases.

To solve this problem, the post array part was combined with the microfluidic device for complete droplet splitting. The splitting behaviors of the microdroplet in the fabricated microfluidic device with the post array part were inspected at 10, 60, and 100 mL/h. hexadecane (with 3 wt.% SPAN80) solution and deionized water were used as the dispersed phase and continuous phase in the O/W system. Microdroplets could be prepared stably by using the proposed microfluidic device combined with the post arrays of all three types. The microdroplet size decreased with increased passes of droplets splitting by the post array part. The device stably worked until 150 mL/h and the resulting solution leaked from the device at higher flow rates than 150 mL/h.

**Figure 7:**
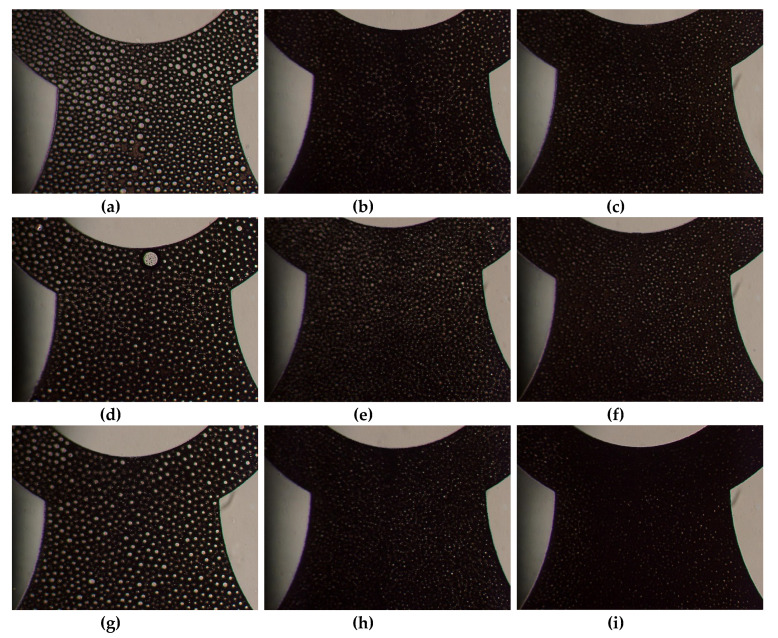

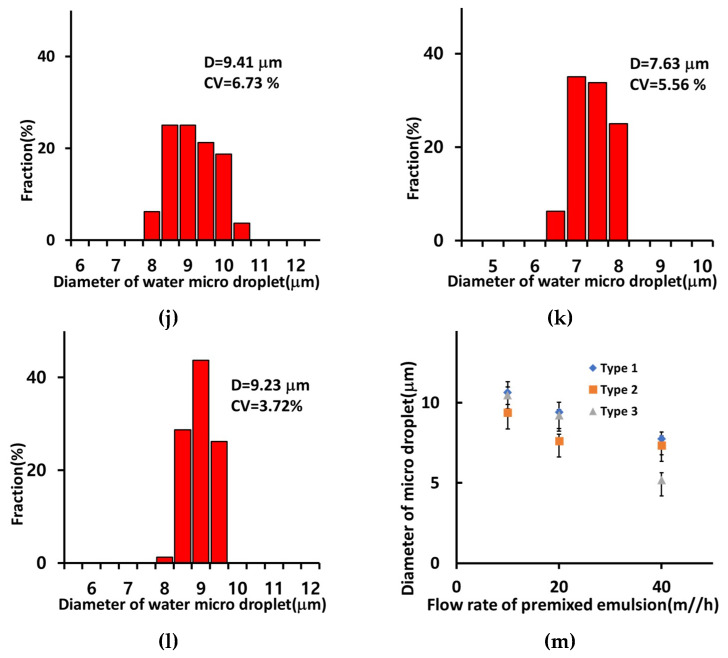
Optical images of microdroplets prepared using 512 channel T-junction passive breakup device combined with a post array part according to the flow rate: 10 mL/h, 60 mL/h, and 100 mL/h: Type 1 (**a**–**c**), Type 2 (**d**–**f**), and Type 3 (**g**–**i**). The graphs of diameters and size distribution of micro water droplets with post array part (Q_mix_ = 20 mL/h): (**j**) Type 1, (**k**) Type 2, and (**l**) Type 3. and (**m**) diameter of water microdroplets according to flow rate of premixed emulsion with post array parts.

Figure 7a–i show the diameter and size distribution of the water microdroplets prepared using the 512-channel geometric droplet-splitting microfluidic device combined with three type of post array. The diameters of the prepared microdroplets were 9.41 µm, 7.63 µm, and 9.23 µm for the Type 1 (Figure 7a–c), Type 2 (Figure 7d–f), and Type 3 (Figure 7g–i) post arrays. The diameter of microdroplets in the type 2 significantly decreased to 81.1% of type 1 because width between posts decreased from 1.25 to 0.75 mm.

Moreover, the size distributions of the microdroplets were 6.73%, 5.56%, and 3.72% for Type 1, Type 2, and Type 3 post arrays. The size distribution of microdroplets can thus be modified using the post array part. The microsphere size distributions improved by 23.5%, 36.8%, and 57.7% when using the Type 1, Type 2, and Type 3 post arrays compared to that without any post array. The size distribution of micro droplet in Type 3 was especially low since it was designed that the streamline at the inlet is perpendicular with a streamline at the post array part to disperse droplets without gathering along the streamline.

In all types of post array parts, the diameter of the microdroplets decreased significantly as the flow rate increased (Figure 7m). The diameters of the microdroplet obtained with the Type 2 post array were smaller than those obtained with the Type 1 post array at 10 mL/h to 40 mL/h because of the narrower distance between posts. The diameter of microdroplets prepared with the Type 3 post array was the smallest among the three types of post arrays under the emulsion flow rate of 40 mL/h due to increased opportunities for droplet splitting by changing flow direction at the inlet.

The diameter of microdroplets could be controlled by varying the flow rate and the design of the post array part. We have demonstrated that the post array has potential for improvement of microdroplets monodispersed. However, the size distribution of the microdroplets was mainly influenced by the inherent premixed emulsion condition. Therefore, the 512-channel geometric droplet-splitting microfluidic device should be studied further regarding the premixed emulsion condition, post array for preparation monodispersed microsphere and minimization of the flow resistance for mass production.

## 4. Conclusions

We presented a 512-channel geometric droplet-splitting microfluidic device for mass production of microspheres. The diameter and size distribution of water microdroplets prepared using the proposed microfluidic device were 35.29 µm and 8.80%, respectively. Additionally, we demonstrated the preparation of biocompatible microspheres by using the proposed microfluidic device. The diameter and size distribution of PLGA microspheres were 6.56 µm and 8.66%, respectively. Additionally, the 512-channel geometric droplet-splitting microfluidic device was combined with a post array part to achieve adequate passes of droplet splitting and stable droplet preparation. Microdroplets were prepared stably at emulsion flow rates of up to 150 mL/h. The three types of post array parts significantly modified the size distribution and diameter of microdroplets. As the post array interval decreased, the size distribution of microdroplets improved. With the Type 3 post array part, more monodisperse microdroplets (CV=3.72%) were obtained than those possible with Type 1(CV=6.73%) and Type 2(CV=5.56%) post array parts. We predict that the proposed device will be considered an alternative approach for the mass production of microspheres in various fields.

## Figures and Tables

**Table 1:** Microchannel width of T-junction in the presented microfluidic device (unit: μm).

**a**	1st T-Junction	500	**b**	2nd T-Junction	540
**c**	3rd T-junction	330	**d**	4th T-junction	200
**e**	5th T-junction	130	**f**	6th T-junction	80
**g**	7th T-junction	50	**h, i**	8, 9th T-junction	30
